# Comprehensive Density Functional Theory Studies of Vibrational Spectra of Carbonates

**DOI:** 10.3390/nano10112275

**Published:** 2020-11-17

**Authors:** Yurii N. Zhuravlev, Victor V. Atuchin

**Affiliations:** 1 Institute of Basic Sciences, Kemerovo State University, 650000 Kemerovo, Russia; zhur@kemsu.ru; 2 Research and Development Department, Kemerovo State University, 650000 Kemerovo, Russia; 3 Laboratory of Optical Materials and Structures, Institute of Semiconductor Physics, SB RAS, 630090 Novosibirsk, Russia; 4 Laboratory of Semiconductor and Dielectric Materials, Novosibirsk State University, 630090 Novosibirsk, Russia

**Keywords:** Density Functional Theory, normal vibrations, infrared spectra, Raman spectra, metal carbonates, cation radius

## Abstract

Within the framework of the density functional theory (DFT) and the hybrid functional B3LYP by means of the CRYSTAL17 program code, the wavenumbers and intensities of normal oscillations of MgCO_3_, CaCO_3_, ZnCO_3_, CdCO_3_ in the structure of calcite; CaMg(CO_3_)_2_, CdMg(CO_3_)_2_, CaMn(CO_3_)_2_, CaZn(CO_3_)_2_ in the structure of dolomite; BaMg(CO_3_)_2_ in the structure of the norsethite type; and CaCO_3_, SrCO_3_, BaCO_3_, and PbCO_3_ in the structure of aragonite were calculated. Infrared absorption and Raman spectra were compared with the known experimental data of synthetic and natural crystals. For lattice and intramolecular modes, linear dependences on the radius and mass of the metal cation are established. The obtained dependences have predictive power and can be used to study solid carbonate solutions. For trigonal and orthorhombic carbonates, the linear dependence of wavenumbers on the cation radius *R*_M_ (or M–O distance) is established for the infrared in-plane bending mode: 786.2–65.88·*R*_M_ and Raman in-plane stretching mode: 768.5–53.24·*R*_M_, with a correlation coefficient of 0.87.

## 1. Introduction

Carbonates form an extensive class of chemical compounds containing the carbonate ion ${\mathrm{CO}}_{3}^{2-}$ as the main structural element [1,2]. In nature, carbonates are found in many minerals and play a crucial role in the carbon exchange of our planet [2,3,4,5,6,7,8]. Carbonate compounds are widely used in the construction industry, optics, and nanotechnology [9,10,11,12,13,14,15]. Appearance of the carbonate species due to chemical interaction with the atmosphere agents was detected at the surface of many oxide materials widely used in optical and electronic technologies [16,17,18,19,20]. In recent years, many artificial crystals of complex carbonates that have no analogues in nature were created [1]. Due to the specific features of the crystal structure, such materials, in many cases, are characterized by high birefringence, nonlinear optical properties, and transparency in the ultraviolet spectral range, which makes them perspective materials for use in optical devices in the UV range [21,22,23,24,25,26,27]. In this aspect, it is of particular importance to study the physicochemical properties of carbonates—in particular, the relationship between their structural and spectroscopic characteristics. According to this approach, this work is aimed at a systematic study of the relationship between the crystal structure and vibrational characteristics of a set of crystals of simple and binary anhydrous carbonates known in nature. Such minerals form several crystal-chemical families, which makes it possible to study the effects of cation substitution on the wavenumbers of vibrational modes within the framework of a single structural type. In the near future, with the accumulation of experimental data of complex carbonates, this algorithm can be extended to new families of complex artificial crystals. 

**Figure 1:**
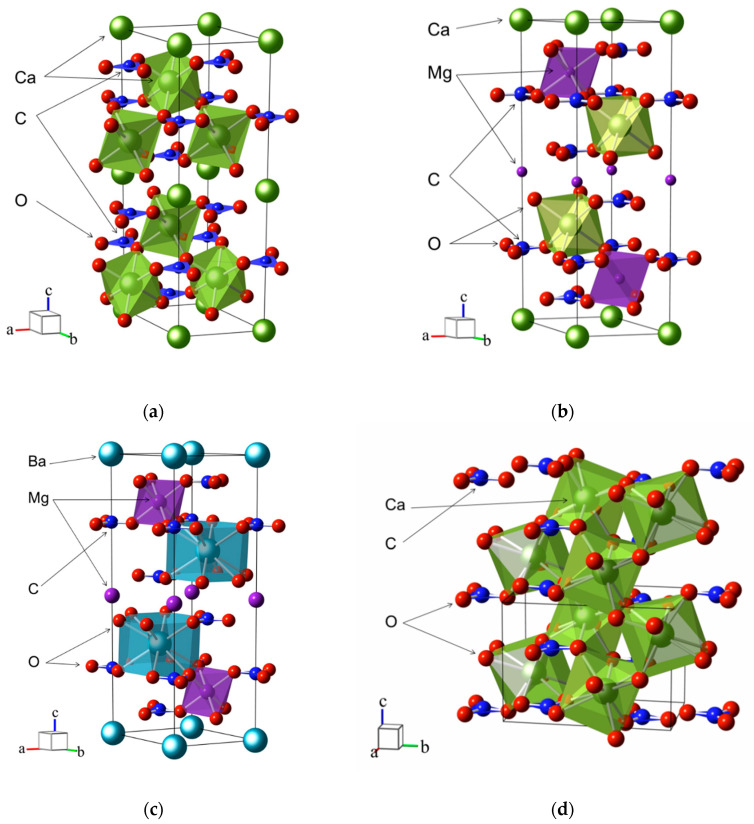
Fragments of the crystal structures of (**a**) CaCO_3_ (calcite), (**b**) CaMg(CO_3_)_2_ (dolomite), (**c**) BaMg(CO_3_)_2_ (norsethite) and (**d**) CaCO_3_ (aragonite) The unit cells are outlined. Lone atoms, excepting those in the unit cells, are omitted for clarity.

Natural carbonates are composed of over 60 minerals [2]. Simple and double rock-forming carbonates can be divided into three main groups based on the similarity of structures: calcite, dolomite, and aragonite. The structures of these crystals are shown in Figure 1. Calcite is the most abundant of all carbonate minerals [28]. The triangular geometry ${\mathrm{CO}}_{3}^{2-}$ dominates in the structure of calcite, which leads to rhombohedral symmetry of the crystal lattice with the space group *R*-3*c*. A primitive cell contains two formula units (*Z* = 2). Divalent cations are octahedrally coordinated by oxygen atoms (Figure 1a). The calcite group includes anhydrous carbonates with the general formula MCO_3_ (M: Ca^2+^, Mg^2+^, Fe^2+^, Zn^2+^, Mn^2+^, Co^2+^, Ni^2+^ and Cd^2+^): calcite (CaCO_3_) [29], magnesite (MgCO_3_) [30], siderite (FeCO_3_) [31], smithsonite (ZnCO_3_) [32], hodochrosite (MnCO_3_) [33], spherocobaltite (CoCO_3_) [34], gaspeite (NiCO_3_) [35], and otavite (CdCO_3_) [36]. The ability to form isomorphic mixtures is widespread among the minerals of the calcite series [37]. 

One of the most common minerals is a double carbonate—dolomite (CaMg(CO_3_)_2_) [38]. Dolomite is structured by natural minerals: minocordite (CaZn(CO_3_)_2_) [39], ankerite (CaFe(CO_3_)_2_) [40], and kutnohorite CaMn(CO_3_)_2_) [41]. Several crystals from the dolomite family were synthesized, including CdMg(CO_3_)_2_, CdMn(CO_3_)_2_, and CdZn(CO_3_)_2_ [42], and the structures were identified in [43]. Under ambient conditions, dolomite crystallizes in a rhombohedral structure with the space group *R*-3 (*Z* = 2). Its layered structure consists of alternating [CaO_6_] and [MgO_6_] octahedra separated by nearly flat and parallel carbonate groups. The structure differs from calcite by the absence of a slip plane. 

Double carbonates are also known in the structure of norsethite (BaMg(CO_3_)_2_) [44] (Figure 1c). BaMn(CO_3_)_2_ does not exist in nature, but it was synthesized in [45]. The norsethite structure is described in *c*-space group *R*-3*c* symmetry with doubled *c*-axis, which corresponds to different rotations of carbonate groups [46]. As the temperature rises in BaMg(CO_3_)_2_, a phase transition is observed from a phase stable under ambient conditions to a high-temperature structure, which is accompanied by a change in the symmetry *R*-3*c* → *R*-3*m*. In the *R*-3*m* symmetry, the unit cell of BaMg(CO_3_)_2_ consists of the [MgO_6_] octahedron, [BaO_12_] polyhedron, and anions. Octahedra and polyhedra are in alternating layers, they are located exactly one above the other, parallel to the [001] direction and are separated by triangular groups ${\mathrm{CO}}_{3}^{2-}$. Natural isostructural orthorhombic carbonates are aragonite (CaCO_3_) (Figure 1d), strontianite (SrCO_3_), cerussite (PbCO_3_), and witherite (BaCO_3_) [47,48], listed in the order of increasing the size of the cation. In a crystal structure with the space group *Pmcn* (*Z* = 4), layers of 9-coordinated cations M^2+^ (M: Ca^2+^, Sr^2+^, Pb^2+^, Ba^2+^) in an approximately hexagonal close packing alternate with layers of planar ${\mathrm{CO}}_{3}^{2-}$ groups arranged perpendicular to the *c*-axis. Like calcites, aragonites form solid solutions [49]. Studies of isostructural orthorhombic carbonates are important for understanding phase transition sequences under pressure and temperature and, therefore, can provide insight into a carbon behavior in the Earth mantle [50,51,52,53].

The optical properties of natural carbonates were extensively explored since these widespread and cheap natural minerals can be used as raw resources for infrared technology materials [54]. In this regard, their infrared spectra (IRS) [55] and Raman spectra (RS) [56,57] were previously studied experimentally and then theoretically [58,59]. Single crystals of iron-free magnesite were studied by Raman spectroscopy [30]; calcite and dolomite crystals were studied in [60] and all crystal were studied in [61]. In [62], they were evaluated with the methods of laser Raman spectroscopy and density functional theory (DFT) calculations using the plane wave basis (PW) and pseudopotentials of the Troullier–Martins type (TM PP). Otavite vibrational spectra were measured in experiment [63]. Eight natural carbonate minerals with a calcite structure were studied using Raman spectroscopy [64]. It was shown that changes in the wavenumber of phonon modes of the *E_g_*(T) symmetry correlate with the distances between the nearest metal and oxygen atoms M–O and the cation ionic radii. Using a graphical approach, the authors developed the spectroscopic Raman model to calculate the ionic radius of a divalent metal cation present in a mineral. 

In [65], studies of the infrared spectra of natural iron-free dolomite were carried out. A combined study of infrared absorption and Raman scattering on a natural dolomite sample CaMg_0.98_Fe_0.02_(CO_3_)_2_ was performed [66]. DFT using exchange correlation potentials in the local density approximation (LDA) and generalized gradient approximation (GGA) in the PW basis with TM PP were used here to interpret the obtained results. The Raman and infrared spectra of cerussite were measured and compared with the spectral characteristics of other minerals of the aragonite family [67]. RS and IRS at high temperature in situ were measured for aragonite, strontianite, cerussite, and witherite at atmospheric pressure [68]. Studies for high and medium temperature infrared absorption and Raman spectroscopy on a synthetic strontianite sample led to the construction of a pressure-temperature phase diagram [69]. In addition, here for the first time, the absorption spectra in the far infrared range were measured for the entire family of aragonite-type carbonates.

The works in which experimental and theoretical spectroscopic studies were performed for a group of carbonates with different structures are of particular interest. Thus, the purpose of the study [70] was to establish the influence of the M^2+^ cation type on the shift of positions of the absorption bands of various anhydrous carbonate minerals from the calcite and dolomite families. In this contribution, it is shown that the position of the minima of absorption bands is unique for each chemical composition of carbonates and can be a diagnostic indicator in mineralogy. A selection of the frequencies of intramolecular modes for a large number of carbonates is also found in [71]. In [72], infrared spectra in the wavenumber range of 70–650 cm^−1^ were presented for 18 common and rare minerals which are quite pure in composition and have a known crystal structure. It is shown that the spectra in the far infrared range of different carbonates from the same structural group have a pronounced similarity, and the observed shifts demonstrate the effect of changing the mass of cations. The vibrational modes of natural minerals—aragonite, calcite, dolomite, magnesite, rhodochrosite, and siderite—that are active in the Raman spectrum were observed, and their pressure and temperature inducing frequency shifts were determined [73]. 

Thus, there are comparatively numerous experimental and individual theoretical studies of the vibrational spectra of carbonates in which the patterns of their changes during the substitution of cations were established for certain types of structures. However, systematic theoretical studies from a unified standpoint, carried out for all types of carbonate structures and having a predictive power in early works, are absent. The aim of this work is to theoretically study the dependences of the characteristics of the infrared and Raman spectra of carbonates crystallizing in the structures of calcite, dolomite, and aragonite on the radii and masses of metal cations. The parameters of vibrational spectra of carbonates were calculated within the framework of a unified approach based on the known experimental data of crystal structure. Furthermore, the results of our calculations were compared with the available experimental and theoretical parameters of the infrared and Raman spectra of carbonates and, on this basis, the general regularities of changes in the vibrational spectra were determined upon substitution of metal cations. Then, the information can be used as an instrument in the analysis of vibrational spectra of new crystalline compounds and solid solutions. The calculated quantitative dependences can also be used in noncontact nondestructive diagnostics of carbonates by spectroscopic techniques. 

## 2. Calculation Method

The research of the ordinary optical properties dependences of metal carbonates were carried out within *ab initio* principles using the Hartree–Fock theory (HF) methods and density functional theory, which are well combined in the CRYSTAL17 program code [74,75]. The hybrid functional B3LYP, which includes the 20% HF exchange with the Becke exchange functional [76] and the LYP correlation functional [77], was used. The basic functions were chosen in the form of a linear combination of localized atomic orbitals of the Gaussian type. We used full-electronic basis sets for carbon, oxygen, magnesium, and calcium atoms from [78] and the gaussian basis sets of double-zeta valence with polarization quality basis set for zinc and cadmium atoms [79,80]. We used pseudopotential basis sets from [81] for strontium and barium, those of [82] for manganese, and those of [83] for lead. 

The reciprocal space was sampled using a Monkhorst–Pack [84] grid with 216 independent *k*-points in the irreducible Brillouin zone for trigonal crystals, and 64 points for orthorhombic crystals. The accuracy of the self-matching procedure was no less than 10^−9^ a.u. (1 a.u. = 27.21 eV). The vibrational frequencies of the lattice atoms were calculated using the FREQCALC procedure [85,86]. The phonon harmonic frequencies *ω*_p_ at the point Г (*k* = 0, the center of the first Brillouin zone) were obtained from the diagonalization of the mass-weighted Hessian matrix of the second derivatives of energy with respect to atomic displacements *u* [87,88]:

(1) $$W_{ai,bj}^{Г}=\frac{H_{ai,bj}^{0}}{\sqrt{M_{a}M_{b}}},H_{ai,bj}^{0}=\left(\frac{\partial^{2}E}{\partial u_{ai}^{0}\partial u_{bj}^{0}}\right)\;$$

where atoms *a* and *b* with masses *M_a_* and *M_b_* are displaced in the unit cell (index 0) from equilibrium positions along the *i*- and *j*-Cartesian directions, respectively. The first order derivatives were calculated analytically, whereas the second order derivatives were obtained numerically. The intensity of IR absorption for the ν-vibration was calculated using the Born effective charge tensor Z*, which characterizes the change in dynamics and the electronic configuration of atom displacement. The relative intensities of the Raman peaks were calculated analytically using the extension scheme of the analytical calculation of IR intensity [89]. The proposed technique was previously used to study the ordinary properties of sulfates [90].

## 3. Crystal Structure

The crystal structure parameters of calcite, dolomite, norsethite, and aragonite types calculated in this work are shown in Table 1. There is a close agreement between the optimized and experimental parameters of the crystal lattice. Thus, the standard deviation $\Delta =\sqrt{\frac{1}{N}\overset{N}{\underset{i=1}{\sum }}{\left(\frac{exp_{i}-theor_{i}}{exp_{i}}\right)}^{2}}$ for *N* = 5 theoretical values from Table 1 and the experimental (exp) value for synthetic magnesite [30] is 1.6%, for natural calcite [29]—1.9%, for smithsonite [32]—1.5%, for synthetic otavite [36]—2.4%, for dolomite [38]—1.5%, and for norsethite [46]—3.6%. For four crystals with aragonite structure, the deviation of three lattice constants and two interatomic distances from experimental data for natural minerals [48] is 2.7%. 

As the physical quantities to describe the regularities of changes in the vibrational properties of carbonates from cationic substitution in the lattice, we used the mass of metal atoms (a.m.u.; Mg—23.985, Ca—39.963, Mn—54.938, Zn—63.929, Sr—87.906, Cd—113.904, Ba—137.905, and Pb—207.977) and the effective Shannon ionic radii [91]. The cationic radii of metals are determined by their electronic structure and depend on the coordination environment. The last filled electron shell of the magnesium ion is 2*p*^6^, and the radius of Mg^2+^ surrounded by six nearest neighbors is 0.72 Å. Similarly, for calcium: 3*p*^6^, 1.00 Å. In zinc and cadmium, the filled shells are 3*d*^10^ and 4*d*^10^, and the radii are 0.74 and 0.95 Å, respectively. In aragonite, each Ca^2+^ ion is already surrounded by nine oxygen atoms and, therefore, its effective radius is 1.18 Å. In the case of strontium and barium, the radii for the 9-fold environment are 1.31 and 1.47 Å, and for 12—1.44, 1.61 Å. The electronic configuration of lead [Xe] 4*f*^14^ 5*d*^10^ 6*s*^2^6*p*^2^ distinguishes it from other elements; therefore, the radius of 9-coordinated Pb^2+^ is 1.35 Å, which is larger than that of strontium, but smaller than that of barium. Transition metals have partially filled 3*d* shells with the number of electrons from 5 to 8, and decreasing radii for Mn^2+^ (0.83 Å), Fe^2+^ (0.78 Å), and Co^2+^ (0.745 Å). Following [33], we write the chemical formula of an arbitrary solid solution as M1*_X_*_1_M2*_X_*_2_M3*_X_*_3_CO_3_, *X*1 + *X*2 + *X*3 = 1. Then, the average radius of the cation is determined as <*R*_M_> = *X*1·*R*_M1_ + *X*2·*R*_M2_ + *X*3·*R*_M3_, where *R*_M1_, *R*_M2_, *R*_M3_ are radii of divalent ions M1^2+^, M2^2+^, M3^2+^. For dolomite, the cation radius is 0.86 Å, and for norsethite—1.165 Å. The average atomic mass of metals is calculated in a similar way.

It is convenient to describe the change in crystal cell parameters or frequencies (intensities) of the vibrational spectra *y* on the radii of cations or their masses *(r*) using the linear dependence *y*(*r*) = *y*_0_ + *y*_1_·*r*, where *y*_0_ is the value of the function at *r* = 0*, y*_1_—derivative of the function *y*, characterizing the rate of change of the corresponding value. The obtained calculated data *y*(*r*_i_), *I* = 1, *N* are approximated by a linear dependence (fit), and the accuracy of this procedure is controlled by the relation: $K=\sqrt{\overset{N}{\underset{i=1}{\sum }}{\left(y_{i}^{fit}-\bar{y^{fit}}\right)}^{2}/\overset{N}{\underset{i=1}{\sum }}{\left(y_{i}^{data}-\bar{y^{data}}\right)}^{2}}$, where the average value $\bar{y}=\frac{1}{N}\overset{N}{\underset{i=1}{\sum }}y_{i}$.

The change in the calculated unit cell volume of carbonates by one formula unit *V/Z* from the cation radii *R*_M_ obeys the linear dependence *V*/*Z*(Å^3^) = 25.22 + 31.54·*R*_M_ with a correlation coefficient of 0.936. The large slope of this dependence of 31.54 Å^2^ indicates that the replacement of the cation is of great importance for carbonates. The indicated dependence with the experimental values of volumes has the form *V*/*Z*(Å^3^) = 22.48 + 36.2482·*R*_M_ (Å^3^) with the coefficient *K* = 0.945. For each individual lattice type, the correlation coefficient is much better: for calcite and aragonite, 0.995, and for dolomite, 0.969. The linear dependence is explained by the fact that the cell volume is weakly related to the structure symmetry but is determined by the stacking of layers of polyhedrons, which depends on the ionic radii of the substitutional atoms. According to Vegard’s law, the unit cell parameters change linearly depending on the composition, and for trigonal crystals, it can be written as: *a*(Å) = 4.008 + 0.959·*R*_M_ (0.941), *c*(Å) = 11.772 + 4.917·*R*_M_ (0.88). Hereinafter, the coefficient *K* is indicated in brackets. The linear dependence for all carbonates is fulfilled for the average distance between metal M and oxygen O: *R*_M–O_(Å) = 1.374 + 1.02·*R*_M_ (0.985) with high accuracy. 

## 4. Vibrational Spectra

The rhombohedral cell of calcite contains ten atoms, and 30 possible vibrational modes can be classified for it, according to irreducible representations of the point group as: Г_tot_ = *A*_1g_(R) + 3*A*_1u_ + 3*A*_2g_ + 3*A*_2u_(IR) + 4*E*_g_(R) + 6*E*_u_(IR). *A*_1g_ and 4*E*_g_ modes are active in Raman spectra (R), 3*A*_2u_ and 5*E*_u_ modes are active in infrared (IR), *A*_1u_ and 3*A*_2g_ modes are spectroscopically inactive, and 1*A*_2u_ and 1*E*_u_ modes are acoustic. Nine translational modes will refer to the symmetry *A*_2g_ + *A*_1u_ + *A*_2u_ + *E*_g_ + 2*E*_u_, six librational modes to *A*_2g_ + *A*_2u_ + *E*_g_ + *E*_u_, and twelve internal vibrations to *A*_1g_ + *A*_2g_ + *A*_1u_ + *A*_2u_ + 2*E*_g_ + 2*E*_u_. Modes of the *A*_2u_ symmetry have polarization ***E***||***z*** and modes of *E*_u_ symmetry have ***E***⏊***z*** polarization. Internal vibrations of *E*_u_ symmetry are of the ν_4_ type (in-plane bending), *A*_2*u*_ modes are of the *ν*_2_ type (out-of plane bending), and *E*_u_ symmetry are of the ν_3_ type of symmetric stretching. In the Raman spectrum, the ν_4_ (in-plane asymmetric stretching) mode has *E*_g_ symmetry, the *ν*_1_ symmetric stretch mode has *A*_1g_ symmetry, and a ν_3_ asymmetric stretch type has *E*_g_ symmetry.

For dolomite structure, the expansion of the vibrational representation according to irreducible representations is Г_tot_ = 4*A*_g_(R) + 6*A*_u_(IR) + 4*E*_g_(R) + 6*E*_u_(IR). Nine translational modes refer to symmetry *A*_g_ + 2*A*_u_ + *E*_g_ + 2*E*_u_, six rotational modes refer to *A*_g_ + *A*_u_ + *E*_g_ + *E*_u_, and 12 internal modes refer to 2*A*_g_ + 2*A*_u_ + 2*E*_g_ + 2*E*_u_. For the norsethite type structure with the space group *R-3m*, the expansion of the vibrational representation is: Г_tot_ = 3*A*_1g_(R) + 2*A*_1u_ +*A*_2g_ + 5*A*_2u_(IR) + 4*E_g_*(R) + 6*E_u_*(IR). For the aragonite orthorhombic structure, the symmetry of the carbonate group decreases to *C_s_*. There will be 60 vibrational modes in total, where 1*B*_1u_ + 1*B*_2u_ + 1*B*_3u_ are acoustic. The vibrational representation is decomposed into irreducible representations as Γ_tot_ = 9*A*_g_ + 6*B*_1g_(R) + 9*B*_2g_(R) + 6*B*_3g_(R) + 6*A*_u_ + 9*B*_1u_(IR) + 6*B*_2u_(IR) + 9*B*_3u_(IR). The *B*_2u_ symmetry modes have polarizations ***E***||***x***(***a***), *B*_3u_—***E***||***y***(***b***), *B*_1u_—***E***||***z***(***c***). There will be 24 internal modes, eight of the ν_4_ and ν_3_ types, and four of the ν_2_ and ν_1_ types. The available experimental and theoretical data on vibration spectra of the carbonates under consideration are summarized in Supplementary Materials [92,93,94,95,96,97,98,99,100,101,102,103,104,105,106,107,108,109,110].

## 5. Optical Spectra of Crystals with a Calcite Structure

**Figure 2:**
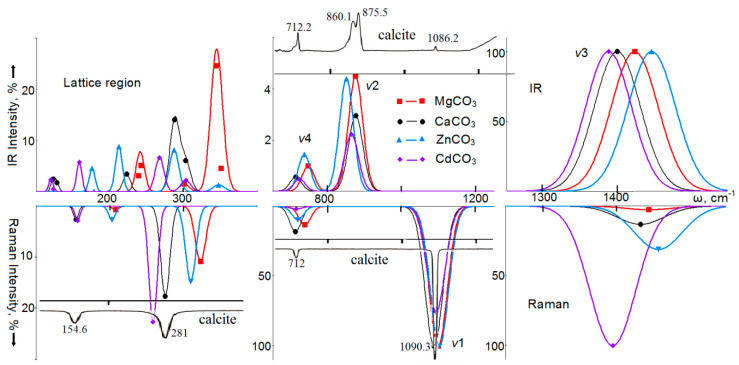
Calculated infrared spectra (IR) (top) and spectra of Raman light scattering (bottom) of intramolecular ν1, ν2, ν3, ν4, and lattice vibrations of magnesium (red, squares), calcium (black, circles), zinc (blue, triangles), and cadmium (lilac, rhombuses) carbonates with a calcite structure. For comparison, the experimental spectra of calcite are given [68].

The infrared absorption spectra (IRS) and Raman scattering spectra (RS) of calcite-structured carbonates calculated in this work, obtained by Gaussian broadening of normal long-wavelength vibrations, are shown in Figure 2. The obtained wavenumbers of vibrations active in the IRS of calcites, together with the available experimental data, are given in Table S1; for the vibrations active in RS, they are given in Table S2 in the Supplementary materials. A good agreement was observed between the calculated vibration wavenumbers and the experimentally measured values. Thus, the average root-square deviations for eight IRS-active wavenumbers obtained by the B3LYP method for magnesite and determined experimentally in [92] and [58] do not exceed 4.0% and 3.3%, respectively. There is also a good agreement with the calculated data of the authors of [93] (4.7%) and [94] (3.5%). The root-mean-square deviations for the wavenumbers of five vibrations active in RS, obtained by the B3LYP method in this work, are 1.3% (1.0%) in the experiment in [95] for magnesite (calcite), 1.5% (1.4%) for the experiment in [58], 1.6% (1.1%) for the experiment in [61], and 1.6% (1.7%) for the experiment in [73]. For four studied carbonates with five vibrations active in RS, in a matrix of 20 values, the deviation of the B3LYP calculation results from the experimental values [64] is 2.4%. 

In MgCO_3_, the most intense mode (5132 km/mol) in IRS corresponds to the internal vibration ν_3_ of *E*_u_ symmetry with the wavenumber of 1424 cm^−1^. Taking its intensity as 100%, for the ν_2_ vibration with a wavenumber of 874 cm^−1^, we obtained 4%, and for the ν_4_ (746 cm^−1^) mode, even less—0.9%. In calcite, the wavenumbers corresponding to the vibrations ν_3_, ν_2_, and ν_4_ are 1400, 875, and 712 cm^−1^, and their intensities are 5447 km/mol (100%), 3%, and 0.5%, which practically do not differ from magnesite. For ZnCO_3_ and CdCO_3_, the structures of the spectra in the high-frequency region remain similar to magnesite. Thus, for internal modes, there is a linear correlation between the change in the wavenumber and the radius of the cation *R*_M_. For ν_4_, it can be written in the form: ω*_E_*_u_(cm^−1^) = 812.9 − 98.7·*R*_M_ (0.958), and with a smaller *K* coefficient for ν_3_: ω*_E_*_u_(cm^−1^) = 1546.9 − 154.8·*R*_M_ (0.854). A good correlation (*K* = 0.96) for the calculated intensity is observed for the ν_3_ vibrations, where it increases with rise of atomic mass as: *I*(km/mol) = 4948 + 14 M, and, for the ν_2_ mode, it decreases with increasing radius: *I*(km/mol) = 478 − 326 *R*_M_.

For lattice vibrations of MgCO_3_, the most intense ones are the *E*_u_ symmetry modes with wavenumbers of 344 cm^−1^ (25%), 301 cm^−1^ (2%), and *A*_2u_ symmetry modes at 351 cm^−1^ (4%), 242 cm^−1^ (5%). For CaCO_3_, the lattice modes are shifted to the low-wavenumber region, and their intensities decrease. In the region of lattice vibrations of ZnCO_3_, the most intense modes will be *E*_u_ symmetry with wavenumbers of 287 cm^−1^, 212 cm^−1^, and only then with *A*_2u_ symmetry: 348 cm^−1^, 176 cm^−1^. In CdCO_3_, this trend continues. Thus, for translation modes of *E*_u_ symmetry, a dependence on the cation mass is observed: ω(cm^−1^) = 306.0 − 1.37·M (0.915). For the rest of the lattice modes, the best linear dependence was established for the cation radius: for rotational ones: ω_Eu_(cm^−1^) = 466.5 − 353.1·*R*_M_ (0.91), ω*_A_*_2u_(cm^−1^) = 463.2 − 348.5·*R*_M_ (0.884, and for translational modes: ω*_A_*_2u_(cm^−1^) = 484.7 − 186.3·*R*_M_ (0.991).

In RS, the intense line (taken as 100%) in Figure 2 is due to vibrations of the ν_1_ type, and falls on 1099 cm^−1^ in MgCO_3_, and 1087 cm^−1^ in calcite. This mode has a significant polarization dependence [89]: the *xx* and *yy* components are ten times larger than the *zz* component. Internal vibrations of ν_4_ type also have a noticeable intensity: for magnesite—with a wavenumber of 737 cm^−1^, for calcite—711 cm^−1^, and also for ν_3_ type: 1444 cm^−1^ and 1433 cm^−1^, respectively. In carbonates of relatively heavy metals zinc and cadmium, the positions of the maxima of the ν_4_ and ν_1_ bands are practically preserved (ω*_E_*_g_(cm^−1^) = 783.3 − 72.9·*R*_M_ (0.906), ω_A1g_(cm^−1^) = 1138.0 − 49.2·*R*_M_ (0.906)), whereas for the ν_3_ region the changes are significant. This is due to the fact that the intensity of this mode increases linearly with an increase in the atomic mass of the metal cation: *I*_ν3_(%) = −29.9 + 1.1·M (0.987). For CdCO_3_, the ν_3_ vibration becomes the most intense in RS and has pronounced *xz* and *yz* polarizations. 

For lattice vibrations, the most intense vibration in the RS spectrum has the *E*_g_ symmetry, and its wavenumbers in MgCO_3_ are 323 cm^−1^ (intensity 11%), ZnCO_3_ 310 cm^−1^ (15%), CdCO_3_ 258 cm^−1^ (23%), and CaCO_3_ 275 cm^−1^ (18%). Thus, for the lattice translational vibration, there is a linear dependence of the form ω(cm^−1^) = 361.0 − 210.6·*R*_M_ (0.992), and for rotational, ω(cm^−1^) = 449.0 − 185.5·*R*_M_ (0.912). Since there is a good linear relationship between *R*_M–O_ and the radius of the *R*_M_ cation, the above formulas can easily be rewritten for distances as well. The above formulas allow predicting the wavenumber values for other carbonates; thus, for the lattice modes *E_g_*(T), *E_g_*(L), internal ν_4_ and ν_1_, the wavenumbers predicted by the formulas for MnCO_3_ are 186, 296, 723, and 1097 cm^−1^, and for CoCO_3_, they are 204, 311, 729, and 1101 cm^−1^. The experimental values for rhodochrosite are 184, 290, 719, and 1086 cm^−1^ [62]; for spherocobaltite, they are 194, 302, 725, and 1090 cm^−1^ [34]. 

## 6. Vibrational Spectra of Crystals with a Dolomite Structure

**Figure 3:**
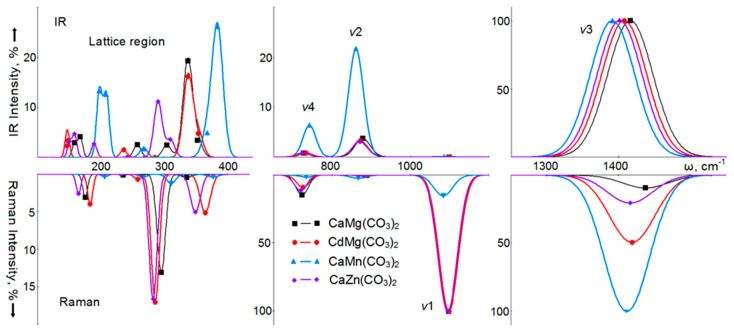
Calculated infrared spectra (IR) (top) and Raman spectra (bottom) of intramolecular ν1, ν2, ν3, ν4, and lattice vibrations of double calcium-magnesium (black, squares), cadmium-magnesium (red, circles), calcium-manganese (blue, triangles), and calcium-zinc carbonates (lilac, rhombuses) with a dolomite structure.

The IRS and RS of carbonates with the dolomite structure are given in Figure 3, and Tables S3 and S4 of the accompanying materials show the wavenumbers of normal long-wave vibrations of crystals with the dolomite and norsethite structures, calculated by the B3LYP method and measured experimentally. The examination of these tables shows that there is a satisfactory agreement between the wavenumbers of lattice [69] and internal [61] vibrations of natural dolomite calculated and measured in IRS. The root-mean-square deviation is 8.7 and 1.0%, respectively. For the wavenumbers of vibrations active in RS, the root-mean-square deviation of the results of this calculation from the experimental values is 1.4%. In crystals with a dolomite structure, the picture of theoretical spectra does not differ significantly from the calcite spectra. In IRS CaMg(CO_3_)_2_, the most intense mode (5318 km/mol, 100%) is the ν_3_ mode at 1416 cm^−1^. The internal vibration ν_2_ with a wavenumber of 877 cm^−1^ has an intensity of 3.5%, and for vibration ν_4_ at 727 cm^−1^, the intensity is close to 1%. Unlike calcite, the vibration ν_1_ of the *A*_u_ symmetry is allowed by symmetry; however, its intensity is practically zero. The most intense (19%) in the region of lattice vibrations is the *E*_u_ symmetry mode with a wavenumber of 337 cm^−1^. Modes of the same symmetry, but with a much lower intensity, appear at 257 (2.5%) and 167 cm^−1^ (4%).

In CdMg(CO_3_)_2_, the most intense (5788 km/mol, 100%) vibration will be ν_3_ with a wavenumber of 1407 cm^−1^, and for lattice vibration with a wavenumber of 338 cm^−1^, the intensity is 16%. A similar picture is observed in CaZn(CO_3_)_2_, where the intensity of the ν_3_ vibration is 5928 km/mol (100%), and the intensities of two lattice vibrations with wavenumbers of 290 and 310 cm^−1^ are 11% and 3.5%, respectively. The situation is different in CaMn(CO_3_)_2_, where the intensity of the ν_3_ mode is much lower—1121 km/mol (100%), and against its background, the relative intensities of other ν_2_ and ν_4_ vibrations increased to 22 and 6%, respectively. 

The RS of dolomite will be dominated by a fully ν_1_ symmetric vibration with a wavenumber of 1097 cm^−1^. Its full intensity is taken as 100%. Then, the intensities of the ν_4_, ν_2_, and ν_3_ modes will be 15%, 0.2% and 9%, respectively. In the region of lattice vibrations, the most intense are the *E*_g_ symmetry modes with wavenumbers of 296 cm^−1^ (13%) and 175 cm^−1^ (3%). In CdMg(CO_3_)_2_, the ν_1_ mode does not change in wavenumber and remains most intense. The wavenumber of the ν_3_ mode decreases, but its intensity sharply increases to 49%. In CaMn(CO_3_)_2_, the intensity of the ν_3_ mode becomes maximum (taken as 100%), while for the ν_1_ vibration it is only 15%. Thus, as for IRS, the binary carbonate CaMn(CO_3_)_2_ differs from other crystalline dolomites in the parameters of its vibrational spectra. 

**Figure 4:**
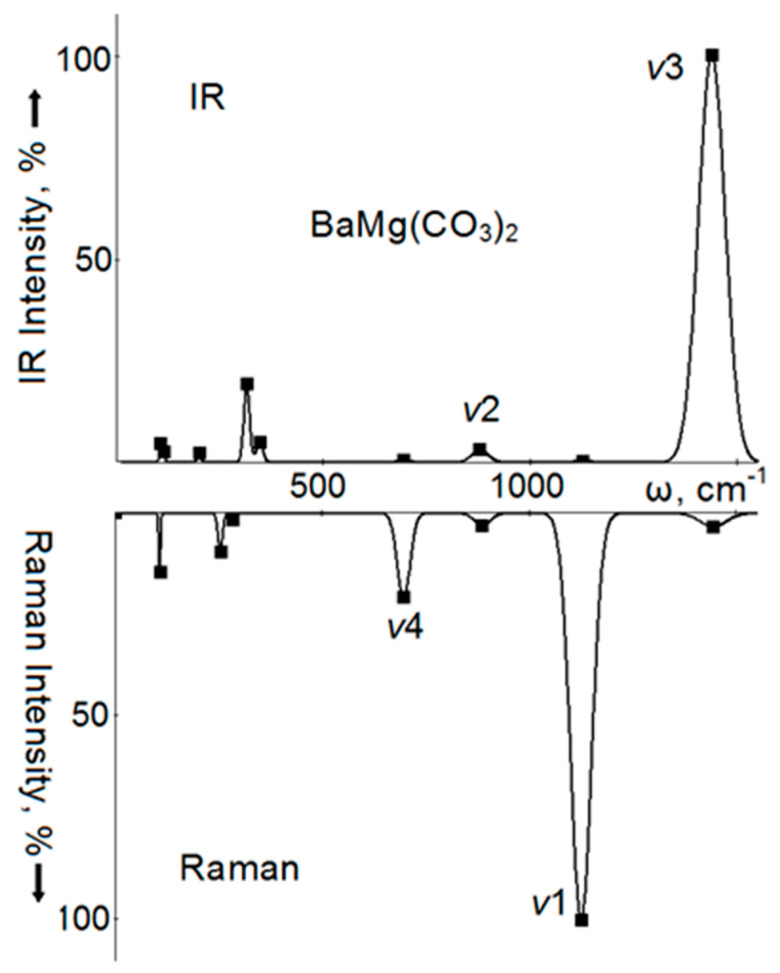
Calculated infrared spectrum (IR) (top) and Raman spectrum (bottom) of intramolecular ν1, ν2, ν3, ν4, and lattice vibrations of double barium-magnesium carbonate in a norsesite-type structure. The solid line is the Gaussian broadening of the frequencies of long-wave oscillations (squares).

In IRS of the BaMg(CO_3_)_2_ crystal (Figure 4) in the region of intramolecular vibrations, the most intense (5194 km/mol) vibration will be ν_3_ with a wavenumber of 1439 cm^−1^. Against this background, the ν_2_ vibration with a wavenumber of 878 cm^−1^ and the intensity of 3% is almost imperceptible, moreover, the ν_4_ vibrations (694 cm^−1^, 0.5%) and ν_1_ allowed here (1125 cm^−1^, 0.2%) practically do not appear. In the region of lattice vibrations, vibrations of *E*_u_ symmetry with wavenumbers of 315, 200, and 106 cm^−1^ stand out in intensity, while less intense vibrations of *A*_2u_ symmetry have wavenumbers of 347 and 115 cm^−1^. The first of these less intense vibrations corresponds to the displacements of magnesium atoms in antiphase with the anions, and the second corresponds to the displacements of barium atoms. Magnesium atoms are also involved in the formation of this mode, and they shift synchronously with the anion. In RS of BaMg(CO_3_)_2_, vibrations of anion atoms will also dominate: ν_1_ with a wavenumber of 1126 cm^−1^ (its intensity is taken as 100%), ν_2_ of the same symmetry and intensity of 3%, as well as doubly degenerated ν_4_ (697 cm^−1^) and ν_3_ (1444 cm^−1^) with intensities of 21% and 3%, respectively. For lattice vibrations, the *A*_1g_ symmetry mode with a wavenumber of 284 cm^−1^ and *E*_g_ symmetry modes with wavenumbers of 108 and 254 cm^−1^ will be noticeable, of which the first is rotational, and the second is translational vibration. 

Let us establish ordinary dependences for the entire class of trigonal crystals. For the lowest- wavenumber lattice translational vibration in IRS ω_Eu_(cm^−1^) = 379.0 − 244.3·*R*_M_ (0.794); for RS, ω_Eg_(cm^−1^) = 367.3 − 216.2·*R*_M_ (0.896), and ω_Eg_(cm^−1^) = 413.1 − 39.6·*R*_M_ (0.813). Using the first formula, we obtain for ankerite (CaFe(CO_3_)_2_) 164 cm^−1^ (in experiment, 166 cm^−1^ [72]), kutnogorite Ca_0.78_Mn_1.13_(CO_3_)_2_ 159 cm^−^^1^ (153 cm^−^^1^). For lattice vibrations active in RS, the formulas give estimated values for rhodochrosite (MnCO_3_) 188, 297 cm^−^^1^. The experimental values are 185 and 290 cm^−^^1^ [73]. 

## 7. Vibrational Spectra of Crystals with Aragonite Structure

**Figure 5:**
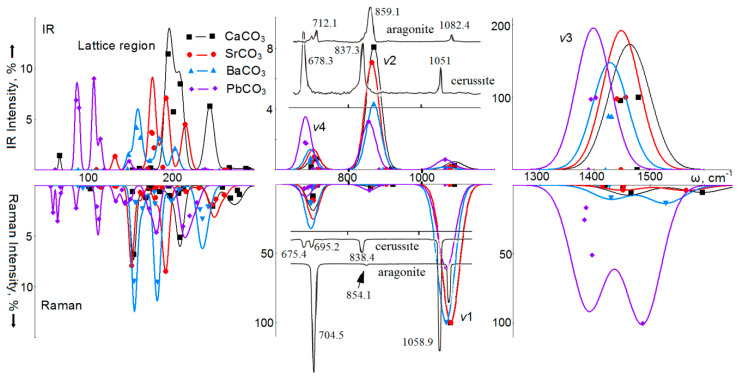
Calculated infrared spectra (IR) (top) and Raman spectra (bottom) of intramolecular ν1, ν2, ν3, ν4, and lattice vibrations of calcium (black, squares), strontium (red, circles), barium (blue, triangles), lead carbonates (lilac, rhombuses) with aragonite structure. For comparison, the experimental spectra of aragonite and cerussite are given [68] (Reproduced with permission from [68]; Copyright Springer Nature, 2020).

The calculated spectra of infrared absorption and Raman light scattering of calcium, strontium, lead, and barium carbonates with aragonite structure are shown in Figure 5. The wavenumbers of normal long-wave vibrations of crystals with the aragonite structure calculated by the B3LYP method, together with the available experimental and theoretical data, are summarized in Tables S5–S8 of the Supplementary Materials. The comparison of wavenumbers calculated by the B3LYP method with the experimental values shows that the root-mean-square deviation for four ν_1_–ν_4_ wavenumbers in four carbonates is, according to [68], 1.5% for IRS; 0.8% for RS; for IRS [55]—2.0%, and for RS [57]—0.9%. 

In the IRS of aragonite, the most intense vibrations are of the ν_3_ type with the *B*_2u_*, B*_3u_ symmetry, wavenumbers of 1448 and 1480 cm^−1^ and intensities of 4523 and 4727 km/mol. In Figure 4, they correspond to a broad intense band with the maximum at 1462 cm^−1^ (1461 cm^−1^ in [68]). For convenience of comparison, the intensity of vibration of *B*_3u_ symmetry is taken as 100%. In SrCO_3_, the maximum intensity of the *B*_3u_ mode at 4757 km/mol is taken as 100%, in BaCO_3_, the *B*_2u_ symmetry modes at 4953 km/mol, and in PbCO_3_: 6241 km/mol. Thus, the intensity ν_3_ increases with the atomic mass of the metal, and the position of the maximum in the series changes according to the law: ω(cm^−1^) = 1480.2 − 0.365·M (cm^−1^) with a correlation coefficient of 0.991. 

Unlike calcite, in the infrared spectra of aragonite, the vibration of the ν_1_ type will be active due to the modes of symmetry *B*_3u_, *B*_1u_. In the spectrum shown in Figure 4, they correspond to a weak (0.2%) band with a maximum at 1090 cm^−1^, the wavenumber of which shifts towards lower values with increasing atomic mass: ω(cm^−1^) = 1090.6 − 0.127·M. The intensity of vibrations of the ν_2_ type of *B*_1u_ symmetry is much higher than that of ν_1_ vibrations, it decreases with an increase in the atomic mass of the metal, and its wavenumber practically does not change: ω(cm^−1^) = 893.3 − 0.065·M. In the experimental spectra, this dependence has the form: ω(cm^−1^) = 866.2 − 0.123·M. Vibrations of the ν_4_ type in CaCO_3_ and SrCO_3_ correspond to the modes of symmetries *B*_3u_*, B*_2u_ with distances between wavenumbers of 13 and 6 cm^−1^. In BaCO_3_ and PbCO_3_, the distances between wavenumbers decrease to 2 and 3 cm^−1^. This behavior of vibrational modes of the ν_4_ type is consistent with experimental data [68], where it was found that two peaks are observed in aragonite (CaCO_3_) and strontianite (SrCO_3_), and only one for cerussite (PbCO_3_) and witherite (BaCO_3_). There is a good ordinary dependence of the peak position on the atomic mass of the metal: in the experiment it is ω(cm^−1^) = 707.5 − 0.128·M, and in the calculation: ω(cm^−1^) = 715.4 − 0.119·M, with the correlation coefficients 0.944 and 0.977, respectively. 

In the Raman spectrum of aragonite (CaCO_3_), ν_1_ vibration of *A*_g_ symmetry with a wavenumber of 1078 cm^−1^ is the most intense (taken as 100%). Vibrations ν_4_ of the *A*_g_ and *B*_1g_ symmetries with intensities of 9% each are also noticeable, which form the maximum in the spectrum shown in Figure 5 at 702 cm^−1^. In BaCO_3_, the ν_1_ vibration maximum shifts to 696 cm^−1^, and its intensity increases. In addition, the width of this peak increases. Oscillations of the ν_3_ type in CaCO_3_ have *B*_3g_ (1465 cm^−1^) and *B*_2g_ (1595 cm^−1^) symmetries, and an intensity of 5%. In SrCO_3_, these are vibrations at wavenumbers of 1450 and 1565 cm^−1^ with intensities of 4%. In BaCO_3_, they shift to lower values of 1429 and 1528 cm^−1^, and the intensities increase to 9 and 13%. Oscillations of the *B*_2g_ symmetry correspond to the displacements of atoms to the C–O bonds along the *b* axis, whereas *B*_3g_—along the *a* axis, perpendicular to the layers of anions and cations.

The Raman spectrum of PbCO_3_ differs from the spectra of other carbonates with the aragonite structure. Here, the most intense (taken as 100%) is the vibration of the ν_3_ type of *B*_2g_ symmetry with a wavenumber of 1486 cm^−1^ [68]. Three other vibrations of this type have *B*_3g_*, A*_g_*, B*_1g_ symmetries, similar wavenumbers of 1394, 1383 and 1380 cm^−1^ and intensities of 50%, 16%, and 25%, respectively. They form a second maximum in RS at 1388 cm^−1^. The ν_1_-type vibration with a wavenumber of 1067 cm^−1^ has an intensity of 61%. Modes of the same *A*_g_ symmetry are also dominant in the formation of the ν_2_ band, the position of which in aragonites obeys the law: ω(cm^−1^) = 895.6 − 0.117·M with a high correlation coefficient of 0.977. 

In the IRS of aragonite, in the region of lattice vibrations, there is a maximum at 245 cm^−1^, formed by the *B*_3u_ symmetry mode with an intensity of 13%, and the main maximum with an intensity of 23% at 194 cm^−1^ (*B*_2u_ symmetry). For SrCO_3_, four peaks are observed at 129, 175, 192 and 215 cm^−1^ with intensities of 3, 18, 15 and 9%, while in BaCO_3_ the intense band is at 157 cm^−1^ *(B*_1u_ and *B*_3u_*)* with a low-wavenumber band at 146 cm^−1^, and high-wavenumber at 171 cm^−1^ shoulders. In PbCO_3_, the main features of the spectrum are shifted to the low-wavenumber region. There are two main peaks at 82 cm^−^^1^ with an intensity of 17%, and a peak at 103 cm^−^^1^ and an intensity of 18%. This structure of the IRS in the lattice region is consistent with the results of measurements [72], where it was found that the broad band at 263 cm^−^^1^ in the structure of aragonite shifts to longer wavelengths with an increase in the atomic number of the metal: up to 227 cm^−^^1^ in strontianite, then up to 205 cm^−^^1^ in witherite, and, finally, up to 136 cm^−^^1^ in cerussite. 

For lattice vibrations of aragonite in RS, there will be three main bands with maxima at 152 cm^−1^, 209 cm^−1^ and 250, 276 cm^−1^. There is a good agreement between the calculated and experimental data [68]. In SrCO_3_, there are two intense bands with maxima at 150 and 191 cm^−1^, formed by the modes of symmetries *B*_3g_, *B*_2g_*,* and a weak band at 252 cm^−1^. In the BaCO_3_ spectrum, the maximum of the first band is at 153 cm^−1^, the second at 181 cm^−1^ with a shoulder at 198 cm^−1^, and the third at 237 cm^−1^. The second maximum is formed by modes with *B*_2g_ symmetry with pronounced *yz* polarization. The spectrum of PbCO_3_ contains a large number of bands of low intensity, not exceeding 6%.

In [68], it was found that RS and IRS confirm the general trend that each of the internal modes is shifted to lower wavenumbers in the following order: aragonite → strontianite → witherite → cerussite. The coefficients of linear interpolation of the wavenumbers of intramolecular (ν_4_, ν_1_, ν_3_) vibrations active in RS for carbonates with the aragonite structure, obtained from experimental data [68] and theoretical calculations using the B3LYP method are summarized in Table 2. In the calculations, each type of vibration was determined as the average of the individual modes, which are shown in Table S9. For lattice vibrations, similar linear dependences are also obtained, as recorded in Table S10.

As a check of the obtained formulas, we will use the available data [49], where the Raman spectra of (CaCO_3_) X1 (SrCO_3_) X2 (BaCO_3_) X3 solid solutions were measured. Thus, in the spectrum of the composition 0.34:0.33:0.33, wavenumbers of 1452, 1086, 711, 273, 192, 155 cm^−1^ were observed. Calculation according to the formulas of Tables S8 and S9 gives: 1466, 1077, 703, 262, 183, and 158 cm^−1^, that is, the root mean square deviation of the calculated and experimentally determined wavenumbers is about 2.7%. 

## 8. Conclusions

In this work, the Hartree–Fock theory and the electron density functional in the form of a hybrid B3LYP functional in the basis of a linear combination of atomic orbitals by means of the CRYSTAL17 program code are used to calculate the structure and normal long-wavelength vibrations of MgCO_3_, CaCO_3_, ZnCO_3_, CdCO_3_ in the structure of calcite, CaMg(CO_3_)_2_, CdMg(CO_3_)_2_, CaMn(CO_3_)_2_, CaZn(CO_3_)_2_—in the structure of dolomite, BaMg(CO_3_)_2_—in the structure of the norsethite type, CaCO_3_, SrCO_3_, BaCO_3_, PbCO_3_—in the structure of aragonite. The analysis of the calculated results and their comparison with the available experimental data shows that the wavenumbers and intensities of individual vibrational modes obey the ordinary laws. For the calcite family, the intramolecular modes of the ν_2_ and ν_3_ types that are active in IRS correlate with the cation radius ω(cm^−1^) = 812.9 − 98.7·*R*_M,_ and ω(cm^−1^) = 1546.9 − 154.8·*R*_M_ with correlation coefficients of 0.958 and 0.854. Vibration of the ν_4_ type is active in RS, where the dependence of its wavenumber on the radius of the metal cation has the form ω(cm^−1^) = 783.3 − 72.9·*R*_M_, and for the most intense ν_1_: ω(cm^−1^) = 1138.0 −42.9·*R*_M,_ with coefficients of correlation 0.906. For the entire class of trigonal crystals (calcite, dolomite, norsethite), the dependence of low- wavenumber lattice vibrations has the form for *E*_u_ symmetry: *E*_u_: ω(cm^−1^) =379.0 − 243.3·*R*_M,_ and *E*_g_: ω(cm^−1^) = 367.3 − 216.2·*R*_M_, ω(cm^−1^) = 413.1 − 139.6·*R*_M_. For carbonates with aragonite structure for the calculated wavenumbers ν_4_, ν_2_, ν_1_, ν_3_, linear dependences with high correlation coefficients are obtained for the atomic mass: ω(cm^−1^) = 714.3 − 0.128·M; ω(cm^−1^) = 895.6 − 0.117·M; ω(cm^−1^) = 1087.1 − 0.116·M; ω(cm^−1^) = 1619.0 − 0.638·M, and for IRS: ω(cm^−1^) = 715.4 −−0.119·M’; ω(cm^−1^) = 893.3 − 0.065·M; ω(cm^−1^) = 1090.6 − 0.127·M; ω(cm^−1^) = 1480.2 − 0.365·M. For trigonal and orthorhombic carbonates, linear dependences of metal substitution were established for the radius of the RM cation (the distance between the metal and oxygen *R*_M–O_) only for the infrared in-plane bending mode ν_4_: ω(cm^−1^) = 786.2 − 65.88·*R*_M_ (ω(cm^−1^) = 881.0 − 67.13·*R*_M–O_), and Raman in-plane asymmetric stretching mode ν_4_: ω(cm^−1^) = 768.5 − 53.24·*R*_M_ (ω(cm^−1^) = 844.2 − 53.83·*R*_M–O_), with a correlation coefficient of 0.87 (0.91). For the rest of the modes, it was not possible to obtain linear dependences with high correlation coefficients. 

Thus, *ab initio* predictions, with a relatively low computational capacity, can reproduce the full vibrational spectra of crystalline compounds of material science interest, and, on the basis of ordinary spectral dependences, predict their features for solid crystalline solutions. The obtained quantitative dependences of the characteristics of vibrational modes can be used in non-contact non-destructive diagnostics of carbonates by optical methods. 

## Figures and Tables

**Table 1:** Calculated lattice constants *a*, *b*, *c*, unit cell volume *V*, and average distances between the atoms of metal M and oxygen O *(R*_M–O_) and carbon C and oxygen (*R*_C–O_).

Carbonate	*a*, Å	*b,* Å	*c*, Å	*V*, Å^3^	*R*_M–O_, Å	*R*_C–O_, Å
MgCO_3_	4.6624	4.6624	15.1891	285.9527	2.1229	1.2857
CaCO_3__C	5.0385	5.0385	17.3168	380.7118	2.3905	1.2878
ZnCO_3_	4.7094	4.7094	15.1297	290.5952	2.1344	1.2973
CdCO_3_	4.9819	4.9819	16.6163	357.1529	2.3312	1.2874
CaMg(CO_3_)_2_	4.8382	4.8382	16.2563	329.5605	2.2571	1.2865
CdMg(CO_3_)_2_	4.8140	4.8140	15.8629	318.3695	2.2253	1.2862
CaMn(CO_3_)_2_	4.8295	4.8295	15.8159	319.4686	2.2302	1.2874
CaZn(CO_3_)_2_	4.8558	4.8558	16.2964	332.7782	2.2648	1.2866
BaMg(CO_3_)_2_	5.0637	5.0637	17.0662	378.9683	2.6811	1.2846
CaCO_3__A	5.0020	8.0175	5.8581	234.9323	2.5604	1.2857
SrCO_3_	5.1469	8.4418	6.1947	269.1522	2.6838	1.2884
BaCO_3_	5.3665	8.9327	6.6847	320.4459	2.8567	1.2910
PbCO_3_	5.2453	8.5723	6.3725	286.5319	2.7451	1.2897

**Table 2:** Linear interpolation coefficients of the wavenumbers of intramolecular (ν_4_, ν_1_, ν_3_) vibrations of the M cation mass for carbonates with aragonite structure, active in RS, obtained from experimental data [68] and theoretical calculations by the B3LYP method. The correlation coefficient is shown in brackets.

Method	*ν*_4_	*ν*_1_	*ν*_3_	*v*_3_
Experiment [68]	713.2 − 0.173·M (0.986)	1090.1 − 0.17·M (0.919)	1495.3 − 0.588·M (0.989)	1597.2 − 0.582·M (0.99)
B3LYP	714.3 − 0.128·M (0.933)	1087.059 − 0.116·M (0.894)	1486.0 − 0.426·M (0.992)	1619.0 − 0.638·M (0.998)

## Supplementary Materials

The following are available online at https://www.mdpi.com/2079-4991/10/11/2275/s1, Table S1: Wavenumbers (cm^−1^) of lattice, translational (T), rotational (L) and internal mode vibrations active in IR spectra (IR), obtained in this work by the B3LYP method, measured experimentally (Exp.) and calculated (Theor.) for carbonates with calcite structure, Table S2: Wavenumbers (cm^−1^) of lattice, translational (T), rotational (L), and internal modes vibrations active in the Raman spectra, obtained in this work by the B3LYP method, measured experimentally [Exp] and calculated [Theor] in the works of other authors for carbonates with calcite structure, Table S3: Wavenumbers (cm^−1^) of lattice and internal modes vibrations active in the IR spectra, obtained in this work by the B3LYP method, measured experimentally [Exp.] and calculated [Theor.] in the works of other authors for carbonates with dolomite and norsethite structure, Table S4: Wavenumbers (cm^−1^) of lattice and internal modes vibrations active in Raman spectra, obtained in this work by the B3LYP method, measured experimentally [Exp.] and calculated [Theor.] in the works of other authors for carbonates with dolomite and norsethite structure, Table S5: Wavenumbers (cm^−1^) of internal modes v_1_, v_2_, v_3_, v_4_ vibrations active in infrared spectra (IRS) calculated by the B3LYP method, measured experimentally [Exp.] and calculated [Theor.] in the works of other authors for crystals with aragonite structure, Table S6: Wavenumbers (cm^−1^) of internal modes v_1_, v_2_, v_3_, v_4_ vibrations active in the Raman spectra, calculated by the B3LYP method, measured experimentally [Exp.] and calculated [Theor.] in the works of other authors for crystals with aragonite structure, Table S7: Wavenumbers (cm^−1^) of lattice vibrations, active in infrared (IR) spectra, calculated by the B3LYP method, measured experimentally [Exp.] and calculated [Theor.] in the works of other authors for crystals with aragonite structure, Table S8: Wavanumbers (cm^−1^) of lattice vibrations active in the Raman spectra, calculated by the B3LYP method, experimentally measured [Exp.] and calculated [Theor.] in the works of other authors for crystals with aragonite structure, Table S9: Values of the coefficients ω_0_ (сm^−1^), ω_1_(сm^−1^/a.m.u.) of linear interpolation of frequencies ω = ω_0_ + ω_1_·М (сm^−1^) by the atomic mass of the metal M intramolecular (ν_4_, ν_2_, ν_1_, ν_3_) modes, active in the Raman and infrared spectra absorption (IR) of carbonates with aragonite structure, obtained from theoretical calculations by the B3LYP method. The correlation coefficient K is shown in parentheses. Table S10: Values of the coefficients ω_0_ (сm^−1^), ω_1_(сm^−1^/a.m.u.) of linear interpolation of wavenumbers ω = ω_0_ + ω_1_·М (сm^−1^) by the atomic mass of the metal M of lattice vibrations active in the Raman spectra of carbonates with aragonite structure obtained from theoretical calculations by the B3LYP method. The correlation coefficient *K* is shown in parentheses.
