# Supplementary material for: Comprehensive Density Functional Theory Studies of Vibrational Spectra of Carbonates
Source: Nanomaterials (Basel). 2020 Nov 17;10(11):2275. doi: 10.3390/nano10112275 (PMC7698329; doi:10.3390/nano10112275)
Supplement: Supplementary file 1 [file nanomaterials-10-02275-s001.pdf]

nanomaterials

Supplementary Materials: Comprehensive DFT Studies of Vibrational Spectra of Carbonates

Yurii N. Zhuravlev [1] and Victor V. Atuchin [2,3,4,*]

[1] Institute of Basic Sciences, Kemerovo State University, Kemerovo, 650000, Russia; zhur@kemsu.ru
[2] Research and Development Department, Kemerovo State University, Kemerovo, 650000, Russia; atuchin@isp.nsc.ru
[3] Laboratory of Optical Materials and Structures, Institute of Semiconductor Physics, SB RAS, Novosibirsk, 630090, Russia
[4] Laboratory of Semiconductor and Dielectric Materials, Novosibirsk State University, Novosibirsk, 630090, Russia
* Correspondence: atuchin@isp.nsc.ru; Tel.: +7 (383) 3308889

Table S1. Wavenumbers (cm^{-1}) of lattice, translational (T), rotational (L) and internal mode vibrations active in IR spectra (IR), obtained in this work by the B3LYP method, measured experimentally (Exp.) and calculated (Theor.) for carbonates with calcite structure.

Method	Lattice modes					Internal modes		
	E_u, (*L*)	A_{2u}, (*L*)	E_u, (*T*)	E_u, (*T*)	A_{2u}, (*T*)	ν_4, (E_u)	ν_2, (A_{2u})	ν_3, (E_u)
$MgCO_3$, IR								
B3LYP	238.8	242.1	300.5	348.8	350.7	745.7	874.5	1423.9
Exp. [55]						749	892	1478
Exp. [92]	227	254	308	355	380	749	885	1446
Exp. [58]	225	230	301	356	362	747	876	1436
Exp. [72]	228	256	306		380			
Theor. [58]	240.9	240.6	298.7	343.1	348.4	744.1	874.8	1429
Theor. [93]	228.08	221.59	299.01	350.96	357.82	759.06	934.22	1477.4
Theor. [94]	227.6	252.7	300.1	333.6	376.0	738.0	862.8	1438.0
$CaCO_3$, IR								
B3LYP	122.3	127.3	222.5	287.7	302.2	711.9	874.7	1400.4
Exp. [55]						712	881	1432
Exp. [58]	102	92	223	297	303	712	872	1407
Theor. [58]	124.9	126.1	219.6	285.9	298.9	711.5	874.4	1400
Exp. [72]	110	98	228		319			
Exp. [99]						712	876	1457
Exp. [68]						712	875	1417
$ZnCO_3$, IR								
B3LYP	178.2	175.6	211.9	286.9	347.5	735.1	849.5	1447.0
Exp. [55]						745	873	1480
Exp. [72]			202	309	360			
$CdCO_3$, IR								
B3LYP	122.5	119.3	158.0	267.1	302.9	722.4	863.0	1388.5
Exp. [55]						724	862	1462
$FeCO_3$, IR								
Exp. [55]						738	869	1470
Exp. [72]			195		230			
$MnCO_3$, IR								
Exp. [55]						728	870	1480
Exp.[72]	166	177	205	308	352			
$CoCO_3$, IR								
Exp. [55]						747	869	1485

Table S2. Wavenumbers (cm^{-1}) of lattice, translational (T), rotational (L), and internal modes vibrations active in the Raman spectra, obtained in this work by the B3LYP method, measured experimentally [Exp] and calculated [Theor] in the works of other authors for carbonates with calcite structure.

Method	Lattice modes		Internal modes		
	E_g, (T)	E_g, (L)	ν4, (E_g)	ν1, (A_{1g})	ν3, (E_g)
		$MgCO_3$, Raman			
B3LYP	208.0	323.2	736.6	1098.9	1444.3
Exp. [56]	213	329	738	1093	1444
Exp. [95]	213	329	738	1094	1444
Exp. [58]	212	332	735	1096	1460
Exp. [61]	213.6	331	738.1	1094.9	1445.8
Exp. [64]	215	333	738	1094	1445
Exp. [73]	214	330	738	1094	1446
Exp. [31]	212	330	738	1093	1445
Theor. [96]	208.4	361.7	737.7	1103.3	1452.6
Theor. [58]	207.4	324.9	736.0	1100.3	1446.9
Theor. [93]	204.21	320.88	750.78	1063.62	1497.58
Theor. [94]	230.5	317.2	735.4	1095.1	1456.8
		$CaCO_3$, Raman			
B3LYP	154.1	275.5	711.4	1087.4	1433.0
Exp. [56]	154	281	711	1085	1434
Exp. [95]	156	281	711	1085	1434
Exp. [58]	156	284	712	1086	1434
Exp. [98]	159.6	287.4	712.6	1087.1	1437.1
Exp. [60]	157	278	715	1088	1440
Exp. [62]	156	282	713	1086	
Exp. [61]	155.5	282	711.8	1086	1435.6
Exp. [64]	154	280	711	1085	1434
Exp. [73]	158	284	713	1088	1438
Exp. [68]			712	1090	1439
Theor. [96]	155.5	273.0	711.4	1094.3	1437.6
Theor. [58]	155.9	276.6	710.9	1088.4	1432.4
Theor. [97]	155.1	276.3	711.0	1089.1	1433.3
Theor. [59]	158.1	286.5	709.8	1094.1	1471.2
		$ZnCO_3$, Raman			
B3LYP	207.6	306.2	723.3	1105.4	1457.4
Exp. [64]	194	303	730	1093	1407
		$CdCO_3$, Raman			
B3LYP	156.3	258.8	713.4	1092.8	1395.3
Exp. [63]	165	275	716	1088	1393
Exp. [64]	159	272	715	1088	1392
		$MnCO_3$, Raman			
Exp. [62]	184	290	719	1086	
Exp. [100]	201	302	723	1100	
Exp. [101]	183	288	717	1084	1414
Exp. [64]	185	292	720	1088	1417
		$CoCO_3$, Raman			
Exp. [64]	200	309	730	1092	1420
Exp. [34]	194	302	725	1090	
		$FeCO_3$, Raman			
Exp. [64]	184	287	735	1087	-
Exp. [98]	174	273	720	1074	1415

Table S3. Wavenumbers (cm^{-1}) of lattice and internal modes vibrations active in the IR spectra, obtained in this work by the B3LYP method, measured experimentally [Exp.] and calculated [Theor.] in the works of other authors for carbonates with dolomite and norsethite structure.

**Method**	**Lattice modes**						**Internal modes**			
	A_u	E_u	E_u	A_u	E_u	A_u	ν_4, (E_u)	ν_2, (A_u)	ν_1,(A_u)	ν_3, (E_u)
$CaMg(CO_3)_2$, IR										
B3LYP	158.5	167.5	257.7	304.4	337.5	352.4	726.5	877.4	1098.0	1415.9
Exp. [55]							730	883	1087	1480
Exp. [72]	160	173	263	322	370	408				
Exp. [61]		177.1		301.1	340		723.9	881.7	1098.1	1442.5
Exp. [66]							730	880		1417
Exp. [69]	145	159	252	320	366	408				
Exp. [88]							728	895		1545
Theor. [58]	158.6	165.3	256.1	302.7	339.4	353.8	726.6	877.6	1096.7	1416.5
Theor. [66]	144	153	287	344	386	393	727	850	1110	1442
$CdMg(CO_3)_2$, IR										
B3LYP	146.2	147.5	236.2	285.2	337.8	354.0	733.0	871.3	1098.4	1407.4
$CaMn(CO_3)_2$, IR										
Exp [66]							716	882		1530
B3LYP	208.7	197.7	267.7	268.6	384.5	368.4	741.5	860.5	1086.1	1390.9
$CaZn(CO_3)_2$, IR										
B3LYP	150.5	158.6	189.5	241.7	290.4	309.8	724.4	870.8	1093.9	1401.0
$BaMg(CO_3)_2$, IR										
Mode		E_u	A_{2u}	E_u	E_u	A_{2u}	E_u	A_{2u}	A_{2u}	E_u
B3LYP		105.7	114.8	200.0	315.4	346.5	693.9	878.0	1124.8	1438.8
Exp. [102]							702	880	1117	1467

Table S4. Wavenumbers (cm^{-1}) of lattice and internal modes vibrations active in Raman spectra, obtained in this work by the B3LYP method, measured experimentally [Exp.] and calculated [Theor.] in the works of other authors for carbonates with dolomite and norsethite structure.

**Method**	**Lattice modes**				**Internal modes**			
	E_g	A_g	E_g	A_g	$v4$, (E_g)	$v2$, (A_g)	$v1$, (A_g)	$v3$,(E_g)
$CaMg(CO_3)_2$, Raman								
B3LYP	175.3	235.0	295.7	335.4	722.7	887.6	1097.2	1437.3
Exp. [56]	175		299	335	724		1097	1441
Exp. [95]	178		300	335	724	880	1097	1439
Exp. [60]	176	258	278	299	715		1088	1440
Exp. [61]	177.1		301.1	340	723.9	881.7	1098.1	1442.5
Exp. [66]	176		301	341	723	878	1096	
Exp. [73]	178	229	301	340	724	881	1098	1442
Theor. [96]	231.2			338.3	724.7	876.5	1104.4	1446.6
Theor. [58]	177.0	235.2	295.5	335.8	722.5	888.2	1101.0	1437.7
Theor. [66]	185	223	323	363	722	858	1109	1461
$CdMg(CO_3)_2$, Raman								
B3LYP	183.1	258.4	286.1	365.1	725.0	882.8	1097.9	1419.0
$CaMn(CO_3)_2$, Raman								
B3LYP	206.1	267.0	311.2	377.5	733.6	866.4	1083.8	1411.6
$CaZn(CO_3)_2$, Raman								
B3LYP	165.4	232.7	282.8	349.5	719.4	879.5	1095.2	1416.3
$BaMg(CO_3)_2$, Raman								
Mode		E_g	E_g	A_{1g}	$v4$, (E_g)	$v2$, (A_{1g})	$v1$, (A_{1g})	$v3$, (E_g)
B3LYP		107.6	254.2	283.8	697.3	886.4	1125.9	1443.6
Exp. [102]				294	701	882	1118	1443
$BaMn(CO_3)_2$, Raman								
Exp. [45]		123.26	248.15		695.33	867.9	1096.0	1418.8

Table S5. Wavenumbers (cm^{-1}) of internal modes v_1, v_2, v_3, v_4 vibrations active in infrared spectra (IRS) calculated by the B3LYP method, measured experimentally [Exp.] and calculated [Theor.] in the works of other authors for crystals with aragonite structure.

Method	*v*4			*v*2		*v*1		*v*3		
	B_{2u}	B_{3u}	B_{1u}	B_{1u}	B_{3u}	B_{3u}	B_{1u}	B_{2u}	B_{1u}	B_{3u}
$CaCO_3$, IRS										
B3LYP	697.7	710.6	717.9	869.7	918.7	1089.8	1090.4	1447.6	1477.7	1480.1
Exp. [55]		703	715	866		1087		1430		1550
Exp. [110]		701.3		858		1086.3		1410.1	1489.5	1569.6
Exp. [87]	699.8	712.4	718.3	852.2	908.8	1082.8	1082.8	1444.5	1466.6	
Exp. [99]		701	713	858						1486
Exp. [68]	699.4	712.1		859.1		1082.4				1461
Theor. [96]	697.1	716.3	709.4	857.0	901.0	10945	1095	1448	1476	1471
Theor. [87]	697.4	712.2	719.2	861.9	913.1	1092.9	1092.9	1445.1	1474.1	1469.9
$SrCO_3$, IRS										
B3LYP	699.8	705.9	716.0	864.4	903.8	1077.3	1077.7	1438.7	1446.3	1455.0
Exp. [55]	701	707		845	863	1074				1496
Exp. [110]	696.6			856.5		1073.7		1406.6	1469.2	1531.1
Exp. [68]	698.5	705.5		855.1		1070.6				1473.2
Exp. [69]	701	702		857	862	1071				
$BaCO_3$, IRS										
B3LYP	694.7	696.5	707.4	869.1	895.3	1065.3	1065.9	1425.6	1420.3	1431.2
Exp. [55]	695	709		845	858	1060				1470
Exp. [103]			693	855	840		1059			1440
Exp. [68]	693.1			854.9			1059.3			1463.4
$PbCO_3$, IRS										
B3LYP	684.0	681.2	702.1	855.5	884.4	1066.1	1065.2	1402.2	1389.3	1393.7
Exp. [55]	670	678		826	840	1053				1450
Exp. [104]	700	678		838			1052	1400	1435	
Exp. [67]	698	670	679	839		1053	1051	1396	1432	1456
Exp. [105]	679			839			1051			1398
Exp. [68]	678.3			837.3			1051			1403.7

Table S6. Wavenumbers (cm^{-1}) of internal modes ν$_1$, ν$_2$, ν$_3$, ν$_4$ vibrations active in the Raman spectra, calculated by the B3LYP method, measured experimentally [Exp.] and calculated [Theor.] in the works of other authors for crystals with aragonite structure.

Method	ν4				ν2		ν1			ν3		
	A_g	B_{1g}	B_{3g}	B_{2g}	A_g	B_{2g}	A_g	B_{2g}	B_{1g}	A_g	B_{3g}	B_{2g}
					$CaCO_3$, Raman							
B3LYP	700.9	702.5	706.1	712.7	859.6	920.1	1078	1089	1417	1450	1465	1592
Exp. [106]	701.3	721	705	717	853	907	1085				1462	1574
Exp. [110]	701.3		705.7	716.5	853		1086				1462	
Exp. [57]	701	721	705	717	853	907	1085			1462		1574
Exp. [67]	701	701	705	716	853		1054				1462	
Exp. 87]	700.6	705.7	704.9	715.8	853.0	908.0	1086	1085	1462		1463	1574
Exp. [97]	700.0		705.0		853.8	918.7	1087				1464	1579
Exp. [107]	705.1		708.7	718.7			1086					
Exp. [73]	701		705	716	853		1085				1463	1576
Exp. [68]			704.5		854.1		1088				1467	1579
Theor. [96]	699	703.4	702.1	712.8	857.7	900	1097	1094	1418	1465	1465	1585
Theor. [87]	701.2	705.5	704.2	714.6	862.8	911.8	1095	1092	1415	1474	1464	1592
					$SrCO_3$, Raman							
B3LYP	703.5	703.6	708.3	713.4	866.6	903.9	1081	1077	1415	1453	1453	1567
Exp. [57]	701			711			1079				1446	1546
Exp. [110]	700.3	696.6		710.0			1073				1446	
Exp. [67]	696	701		710	853		1072					
Exp. [108]	699	703		709			1070				1467	
Exp. [68]	699						1072				1446	1543
Theor. [108]	701	707					1069				1467	
					$BaCO_3$, Raman							
B3LYP	694.1	696.3	700.3	702.8	870.2	893.9	1066	1064	1405	1426	1429	1528
Exp. [57]	691						1059				1420	1506
Exp. [67]	689	710		699	847		1060					
Exp. [103]				693	840	855	1059				1440	
Exp. [68]	691.7						1061				1422	1510
					$PbCO_3$, Raman							
B3LYP	679.9	677.6	687.9	697.0	856.0	883.6	1068	1064	1380	1384	1395	1487
Exp. [104]	674	682		695	838		1054			1374	1425	1477
Exp. [57]	673	682		696	839		1054	1063		1372	1418	1474
Exp. [67]	668	681	673	694	837		1054					
Exp. [109]	671. 2	696.6	675.8	683.8	839.2		1055			1371	1421	1478
Exp. [68]	675.4	695.2			838.4		1059			1368	1424	1481

Table S7. Wavenumbers (cm^{-1}) of lattice vibrations, active in infrared (IR) spectra, calculated by the B3LYP method, measured experimentally [Exp.] and calculated [Theor.] in the works of other authors for crystals with aragonite structure.

**Method**	***B*$_{2u}$**			***B*$_{3u}$**				*B*$_{1u}$			
	**$CaCO_3$, IR**										
**B3LYP**	**61**	**159**	**194**	**150**	**200**	**245**	293	173	209	270	290
Exp. [72]	110 (w), 215(s), 263(bs)										
Exp. [87]	105	164	220	144	209	250	298	183	208	259	287
Theor. [96]	53	154	187	171	191	262	285	143	187	232	291
Theor. [87]	65	159	198	147	201	246	293	174	210	269	289
	$SrCO_3$, IR										
B3LYP	129	169	192	106	175	215	263	174	177	188	267
Exp. [72]	140(w), 180(m), 210 (s) 227(s), 267(m)										
	$BaCO_3$, IR										
B3LYP	146	172	184	83	160	203	236	144	155	166	257
Exp. [72]	137(m), 155(m), 182(s), 205(s), 230 (s)<br>*Note:* S = Strong, M = Medium, W = Weak, Sh = Shoulder, b = broad										
	$PbCO_3$, IR										
B3LYP	81	111	153	55	103	146	239	84	115	160	241
Exp. [72]	230(w), 175(s), 210(m),276(m), 315(m)										

Table 8. Wavenumbers (cm^{-1}) of lattice vibrations active in the Raman spectra, calculated by the B3LYP method, experimentally measured [Exp.] and calculated [Theor.] in the works of other authors for crystals with aragonite structure.

**Method**	***B*$_{3g}$**				***B*$_{1g}$**				***B*$_{2g}$**					*A*$_g$				
	**$CaCO_3$, Raman**																	
B3LYP	98	152	195	213	96	167	176	271	184	209	249	261	280	150	171	196	207	282
Exp. [57]	112	152						272		206	248			142	161		214	284
Exp. [49]						155			180	206								282
Exp. [89]	117	156	211	227	126	181	193	278	185	213	253	267	284	144	166	199	219	291
Exp. [107]	113	152					191	274	182	208	252	262		144			216	285
Exp. [73]	125		115	155			192	274	182	208	250	263		145	164	216		285
Exp. [68]	115	153					190	273				257				213		
Theor. [96]	93	150	194	209	94	157	175	265	169	206	245	253	273	145	156	184	190	274
Theor. [89]	97	152	199	213	101	168	178	271	183	207	249	260	279	149	162	196	205	280
	$SrCO_3$, Raman																	
B3LYP	101	148	187	217	111	160	178	252	130	181	190	232	249	112	128	160	179	262
Exp. [57]								246		181		216				148		260
Exp. [49]		148		214				258		180		236	245					
Exp. [108]	100	146		213			170	258		182	194	234	244	113	128		179	
Exp. [68]	101	148		215				260					243	114			180	
	$BaCO_3$, Raman																	
B3LYP	83	152	178	207	89	155	177	233	101	132	181	198	236	85	96	154	163	244
Exp. [57]						155		225			180				136		161	
Exp. [49]						153		225			180				135			
Exp. [68]						156		225			182			90	136			
	$PbCO_3$, Raman																	
B3LYP	49	109	141	171	52	107	137	220	62	108	148	180	230	58	62	81	130	213
Exp. [104]		102				92		245				173	225			123	148	
Exp. [57]						90	131	245				176	219			122	150	199
Exp. [109]							130					173	219			115	148	
Exp. [68]		102					133	244				177	215				153	

Table S9. Values of the coefficients ω_0 (cm^{-1}), ω_1(cm^{-1}/a.m.u.) of linear interpolation of frequencies $\omega=\omega_0+\omega_1\cdot M$ (cm^{-1}) by the atomic mass of the metal M intramolecular (ν_4, ν_2, ν_1, ν_3) modes, active in the Raman and infrared spectra absorption (IR) of carbonates with aragonite structure, obtained from theoretical calculations by the B3LYP method. The correlation coefficient *K* is shown in parentheses.

**IR.**	ν_4			ν_2	ν_1		ν_3		
	B_{2u}	B_{3u}	B_{1u}	B_{3u}	B_{3u}	B_{1u}	B_{2u}	B_{1u}	B_{3u}
ω_0, cm^{-1}	703.6	720.9	723.1	919.1	1090.0	1091.2	1447.6	1491.4	1501.7
ω_1,cm^{-1} a.m.u.	−0.07	−0.182	−0.105	−0.132	−0.122	−0.131	−0.097	−0.472	−0.526
(*K*)	(0.894)	(0.987)	(0.98)	(0.872)	(0.819)	(0.855)	(0.749)	(0.986)	(0.999)

**Raman**	ν_4				ν_2	ν_1	ν_3			
	A_g	B_{1g}	B_{3g}	B_{2g}	B_{2g}	B_{2g}	B$_{1g}$	A_g	B_{3g}	B_{2g}
ω_0, cm^{-1}	710.6	713.2	714.5	718.9	925.45	1091.5	1430.9	1477.7	1486.0	1619.0
ω_1,cm^{-1}/a.m.u.	−0.135	−0.154	−0.117	−0.105	−0.212	−0.152	−0.225	−0.418	-0.426	-0.638
(*K*)	(0.918)	(0.919)	(0.918)	(0.944)	(0.98)	(0.911)	(0.951)	(0.941)	(0.992)	(0.998)

Table S10. Values of the coefficients ω_0 (cm^{-1}), ω_1(cm^{-1}/a.m.u.) of linear interpolation of wavenumbers $\omega=\omega_0+\omega_1\cdot M$ (cm^{-1}) by the atomic mass of the metal M of lattice vibrations active in the Raman spectra of carbonates with aragonite structure obtained from theoretical calculations by the B3LYP method. The correlation coefficient *K* is shown in parentheses.

**Raman**	B_{3g}	B_{1g}		B_{2g}			A_g			
**ω_0, cm^{-1}**	**167.9**	**188.5**	**280.1**	**231.6**	**257.5**	202.5	224.3	222.5	298.5	165.0
ω_1,cm^{-1}/a.m.u.	−0.236	−0.348	−0.305	−0.625	−0.553	−0.703	−0.647	−0.445	−0.407	−0.539
(*K*)	(0.816)	(0.917)	(0.984)	(0.98)	0.945)	(0.983)	(0.964)	(0.995)	(0.999)	(0.986)
